# High-titer preparation of *Bombyx mori *nucleopolyhedrovirus (BmNPV) displaying recombinant protein in silkworm larvae by size exclusion chromatography and its characterization

**DOI:** 10.1186/1472-6750-9-55

**Published:** 2009-06-12

**Authors:** Tatsuya Kato, Suganthi Lavender Manoha, Shigeyasu Tanaka, Enoch Y Park

**Affiliations:** 1 Laboratory of Biotechnology, Faculty of Agriculture, Shizuoka University, 836 Ohya, Suruga-ku, Shizuoka 422-8529, Japan; 2 Integrated Bioscience Section, Graduate School of Science and Technology, Shizuoka University, 836 Ohya, Suruga-ku, Shizuoka 422-8529, Japan

## Abstract

**Background:**

Budded baculoviruses are utilized for vaccine, the production of antibody and functional analysis of transmembrane proteins. In this study, we tried to produce and purify the recombinant *Bombyx mori *nucleopolyhedrovirus (rBmNPV-hPRR) that displayed human (pro)renin receptor (hPRR) connected with FLAG peptide sequence on its own surface. These particles were used for further binding analysis of hPRR to human prorenin. The rBmNPV-hPRR was produced in silkworm larvae and purified from its hemolymph using size exclusion chromatography (SEC).

**Results:**

A rapid method of BmNPV titer determination in hemolymph was performed using quantitative real-time PCR (Q-PCR). A correlation coefficient of BmNPV determination between end-point dilution and Q-PCR methods was found to be 0.99. rBmNPV-hPRR bacmid-injected silkworm larvae produced recombinant baculovirus of 1.31 × 10^8 ^plaque forming unit (pfu) in hemolymph, which was 2.8 × 10^4 ^times higher than transfection solution in Bm5 cells. Its purification yield by Sephacryl S-1000 SF column chromatography was 264 fold from larval hemolymph at 4 days post-injection (p.i.), but 35 or 39 fold at 4.5 or 5 days p.i., respectively. Protein patterns of rBmNPV-hPRR purified at 4 and 5 days were the same and ratio of envelope proteins (76, 45 and 35 kDa) to VP39, one of nucleocapsid proteins, increased at 5 days p.i. hPRR was detected in only purified rBmNPV-hPRR at 5 days p.i..

**Conclusion:**

The successful purification of rBmNPV-hPRR indicates that baculovirus production using silkworm larvae and its purification from hemolymph by Sephacryl S-1000 SF column chromatography can provide an economical approach in obtaining the purified BmNPV stocks with high titer for large-scale production of hPRR. Also, it can be utilized for further binding analysis and screening of inhibitors of hPRR.

## Background

Baculoviruses are large enveloped viruses with double-stranded circular DNA genomes and have been used for various biotechnological applications. Baculoviruses are utilized for the high level production of recombinant proteins in insect cells [1,2] and the gene transduction to mammalian cells both *in vivo *and *in vitro *as a foreign gene delivery vectors [3-5]. Moreover, budded baculoviruses are applied for displaying the recombinant proteins on their surface for antibody production, functional analysis of receptors and vaccine production [5-7]. More recently, an increasing number of investigators have challenged the use of baculovirus for gene therapy applications [8]. The preparation of modified baculovirus vectors which are able to direct gene expression in mammalian cells represents a safer alternative over classical mammalian viruses. In order to use baculoviruses in gene therapy, the development of efficient production process towards high-titer preparation is required, because of low baculovirus transduction efficiency in mammalian cells compared to other viral delivery system, e.g. retroviruses.

*Autographa californica *multiple nucleopolyhedrovirus (AcMNPV) and *Bombyx mori *nucleopolyhedrovirus (BmNPV) have been used in baculovirus expression system. BmNPV especially infects silkworm larvae and has been used for large-scale production of recombinant protein economically because there is no necessity for cell cultivation [9-11]. Moreover, it is very difficult to cultivate Bm cells with a suspension culture and amplify BmNPV in Bm5 cell culture. The hemolymph of baculovirus-infected silkworm larvae is used as a high titer-baculovirus solution. For medical or biological uses, baculovirus purification using cation-exchange chromatography [12], size exclusion chromatography (SEC) [13], and ion-exchange membrane chromatography [14] from insect cell culture supernatant have been reported until now. But to date, the purification of baculovirus from silkworm larval hemolymph has neither been performed nor reported.

In this study, rBmNPV-hPRR, which displays a native form of human prorenin receptor (hPRR) with FLAG peptide sequence behind its signal peptide sequence on its own surface [15], was produced in silkworm larval hemolymph; purified by ultracentrifugation and two different types of SEC. In addition, the rapid method of BmNPV titer measurement was established using quantitative real-time PCR (Q-PCR). This is the first report of recombinant protein-displayed BmNPV purified from silkworm larval hemolymph by purification with the help of SEC.

## Results

### Establishment of BmNPV titer measurement by Q-PCR

Until now, plaque assay [16], end-point dilution method [17] and antibody-based assay [18] have been known as titer determination method for baculoviruses. The antibody-based assay allows the time reduction required for baculovirus titer, but the antibody for a baculovirus-specific protein, DNA-Binding Protein (DBP) which is expensive is mandatory. Recently, the plotting of cross points for individual baculovirus DNA dilution measured by Q-PCR and titer determination by end-point dilution method has a precise linear correlation with baculovirus titer [19]. Therefore, at the first stage of experiments, it was uncertain whether Q-PCR can be applied to BmNPV.

**Figure 1:**
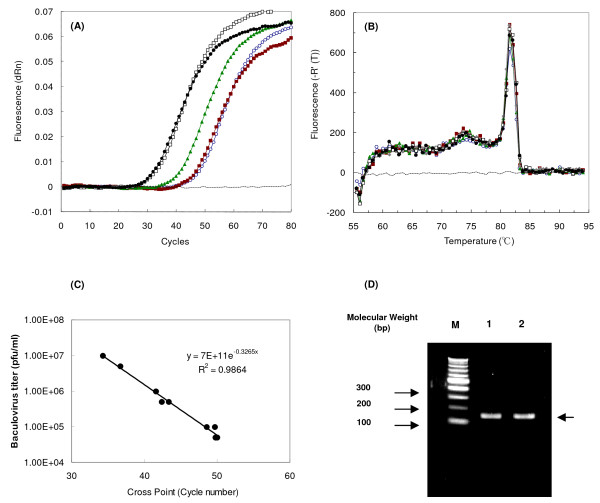
**Q-PCR analysis of BmNPV-*CP*^-^-/bx-GGT2**. (A) The graph of fluorescent signals during Q-PCR cycles using various baculovirus stocks. (B) Melting curve analysis of Q-PCR products. Symbols in A and B: dotted lines, water; open circles, 4.9 × 10^4 ^pfu/ml; closed squares, 9.8 × 10^4 ^pfu/ml: closed triangles, 4.9 × 10^5 ^pfu/ml; closed circles, 4.9 × 10^5 ^pfu/ml; open squares, 9 × 10^5 ^pfu/ml. (C) Standard calibration curve of cross point vs. log (BmNPV-*CP*^-^/bx-GGT2 titer). (D) Analysis of Q-PCR products using BmNPV-*CP*^-^/bx-GGT2 stock solution by 2% agarose gel. Lane M: 100-bp ladder DNA marker, lane 1: stock solution of 9.80 × 10^5 ^pfu/ml, lane 2: stock solution of 9.80 × 10^6 ^pfu/ml.

To apply the Q-PCR on BmNPV titer determination, 142 bp DNA fragment of BmNPV was amplified at the same position, as done for 142 bp DNA fragment of AcMNPV, upstream of ie-1 gene. These two DNA fragments have approximately 95.8% sequence identity. Recombinant BmNPV-*CP*^-^/bx-GGT2 stock [15], whose titer was determined by end-point dilution method, was diluted and its baculovirus DNA was extracted from each diluted baculovirus DNA solution by High Pure Viral Nucleic Acid Kit (Roche Diagnostics K. K.). The cross point for each elute, containing baculovirus DNA, was determined by Q-PCR. As the number of cycle increased, the fluorescent intensity increased in all samples except for a negative control (Figure 1A). No DNA amplification was observed for negative control containing 6 μl of water instead of baculovirus DNA. The melting curves of amplified DNA fragment by Q-PCR were analyzed. Single peaks were observed at approximately 82°C, indicating that DNA fragments were amplified specifically by forward and reverse primers without any unspecific binding (Figure 1B). Standard calibration curve of cross points vs. virus titer is shown in Figure 1C. This result confirms that corresponding cross points and determined titers were correlated linearly with a correlation efficiency of 0.99. On 2% agarose gel, DNA fragment at between 100 and 200 bp markers was found without any other bands (Figure 1D).

### Comparison of baculovirus production using between Bm5 cells and silkworm larvae

For protein expression in Bm5 cells, recombinant BmNPV was prepared by transfection of recombinant BmNPV bacmid DNA into Bm5 cells. Following transfection, BmNPV is required to be amplified several times in order to obtain sufficient virus titer for protein expression. Moreover, time to amplify BmNPV is required due to slow growth rate of Bm cells compared to Sf-9 cells.

Transfection solution was prepared using 13.6 μg of rBmNPV-hPRR bacmid including helper plasmid. Five milliliter of transfection solution was obtained from 2.5 × 10^6 ^Bm5 cells with cultivation after 6 days post-transfection. Hemolymph was also prepared from 13.6 μg of rBmNPV-hPRR bacmid (including helper plasmid)-injected silkworm larva. Approximately 0.55 ml of hemolymph was obtained from a silkworm larva at 6 days p.i. of rBmNPV-hPRR bacmid. Baculovirus titers of transfection solution and BmNPV-infected silkworm larval hemolymph were measured by Q-PCR (Table 1). Titers of transfection solution and hemolymph were 9.34 × 10^2 ^and 2.38 × 10^8 ^pfu/ml, whereas the total baculovirus numbers were 4.67 × 10^3 ^and 1.31 × 10^8 ^pfu, respectively. The baculovirus number from hemolymph was 2.8 × 10^4 ^times higher than that obtained from transfection solution in spite of the use of the same amount of rBmNPV-hPRR bacmid.

**Table 1:** Titer determination of transfection solution and hemolymph from a silkworm larva injected with rBmNPV-hPRR bacmid

	Volume (ml)	Titer (pfu/ml)	Baculovirus number (pfu)
Transfection solution	5	9.34E+02	4.67E+03
Hemolymph	0.55	2.38E+08	1.31E+08

### Baculovirus titer in larval hemolymph from rBmNPV-hPRR-infected silkworm larvae

**Figure 2:**
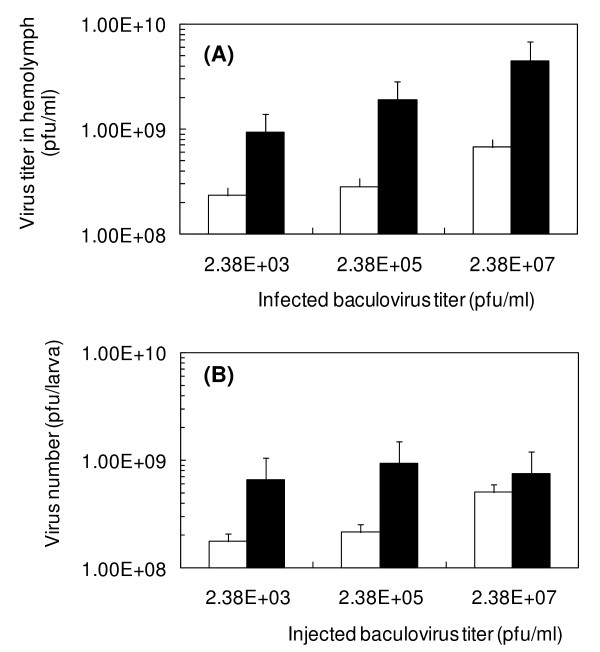
**Virus titer (A) and virus number (B) of hemolymph from silkworm larvae infected with various titers of baculovirus**. Before injection into silkworm larvae, 2.38 × 10^3^, 2.38 × 10^5^, 2.38 × 10^7 ^pfu/ml of baculovirus solution was prepared by dilution of hemolymph from rBmNPV-hPRR bacmid-injected silkworm larvae with PBS (pH 6.2). White and black bars in (A) and (B) indicate 4 and 5 days p.i., respectively.

It was previously reported that baculovirus-infected silkworm larval hemolymph was infectious to silkworm larvae [20]. Thirty μl of rBmNPV-hPRR solutions (2.38 × 10^7^, 2.38 × 10^5^, 2.38 × 10^3 ^pfu/ml) were injected into silkworm larvae, followed by measuring the baculovirus titer after 4 or 5 days post-injection (p.i.). Surprisingly, 72 pfu/larva (30 μl of rBmNPV-hPRR solution containing 2.38 × 10^3 ^pfu/ml) was enough to infect all silkworm larvae. Even the 1:10^5^-diluted hemolymph was still found to be infectious to silkworm larvae. When 30 μl of 2.38 × 10^7 ^pfu/ml rBmNPV-hPRR (7.2 × 10^3 ^pfu/larva) was injected, the highest baculovirus titer (4.50 × 10^9 ^pfu/ml) was obtained for 5 days breeding (Figure 2A). The volume of hemolymph from silkworm larvae injected with 2.38 × 10^7 ^pfu/ml (7.2 × 10^5 ^pfu/larva) at 4 days breeding was similar, but at 5 days breeding it was only one third or one quarter of hemolymph. Therefore, when 2.38 × 10^5 ^pfu/ml baculovirus solution was injected, total baculovirus number in hemolymph was found to be the highest (9.45 × 10^8 ^pfu/larva) (Figure 2B).

### Purification of rBmNPV-hPRR from silkworm larval hemolymph using size exclusion chromatography

Hemolymph was harvested from rBmNPV-hPRR containing hemolymph (approximately 10^5 ^pfu/ml)-injected silkworm larvae at 4 days p.i. and centrifuged at 20000 × g to remove insoluble materials. The supernatant was recovered and centrifuged at 114000 × g in 25% sucrose cushion. The pellet contained a large amount of dark-brownish materials that were hard and insoluble (data not shown). This dark-brownish pellet was obviously different from translucent white baculovirus pellets. In order to remove insoluble substances, the hemolymph was centrifuged again at 20000 × g and filtered using 0.2 μm membrane filters. This filtrate was centrifuged at 114000 × g in 25% sucrose cushion, but still the dark-brownish pellets were visible.

**Figure 3:**
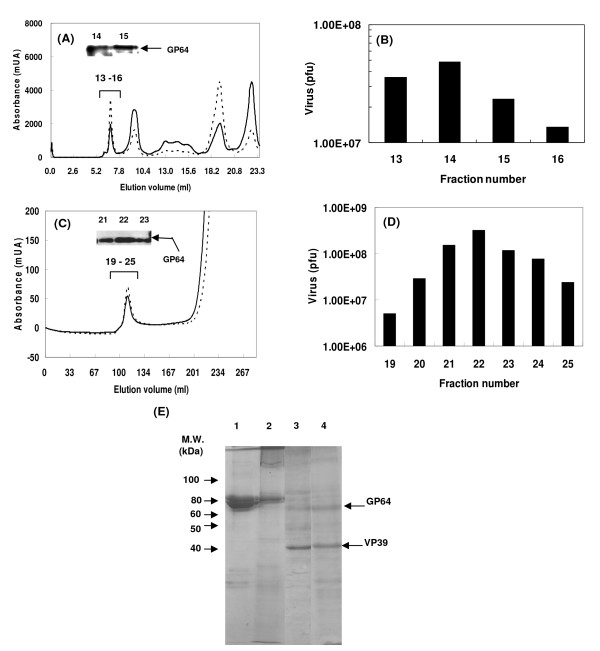
**Purification of rBmNPV-hPRR from hemolymph by Superdex 200 10/300 GL column chromatography and Sephacryl S-1000 SF column chromatography**. (A) and (C) Chromatogram of Superdex 200 10/300 GL and Sephacryl S-1000 SF column chromatography and GP64 detection by Western blot using anti-Bmgp64 antibody. Solid and dotted lines indicate absorbance at 280 and 254 nm, respectively. Numbers on GP64 bands indicate fraction numbers. (B) and (D) Baculovirus number as present in each fraction of Superdex 200 10/300 GL and Sephacryl S-1000 SF column chromatography. (E) SDS-PAGE analysis of proteins of each purified rBmNPV-hPRR fraction stained with CBB. Lane 1, hemolymph; lane 2, rBmNPV-hPRR purified by Superdex 200 10/300 GL column chromatography; lane 3, rBmNPV-hPRR purified by Sephacryl S-1000 column chromatography; lane 4, rBmNPV-hPRR purified from Bm5 cell culture supernatant.

Superdex 200 10/300 GL column was used for purification of rBmNPV-hPRR from larval hemolymph. Larval hemolymph was filtered with 0.2 μm membrane filter, and 2.38 × 10^8 ^pfu/ml rBmNPV-hPRR was obtained. Five hundred μl of the filtrate was applied upon Superdex 200 10/300 GL column equilibrated with PBS. Elution was performed by PBS and every 0.5 ml was collected. Its chromatogram is shown in Figure 3A. The first peak was eluted between 6.5 and 8.5 ml in the void volume, and GP64 was detected in the first peak fractions (Fraction 14, 15 in Figure 3A). Maximum baculovirus titer was 4.86 × 10^7 ^pfu (Figure 3B) at the fraction 14 and the total number of recovered rBmNPV-hPRR was 1.21 × 10^8 ^pfu with recovery ratio of 100% (Table 2). It suggested that there is no loss of BmNPV at this purification step. However the concentration of the virus was done by ultracentrifugation, and its recovery decreased by 30% (Table 2). Finally, rBmNPV-hPRR was purified by 48-fold.

**Table 2:** Summary of purification of rBmNPV-hPRR from hemolymph by Superdex 200 10/300 GL and Sephacryl S-1000 SF column chromatography

	Volume (ml)	Titer (pfu/ml)	Virus (pfu)	Protein (mg/ml)	Virus/Protein (pfu/mg)	Recovery (%)	Purification (fold)
Hemolymph	0.5	2.38E+08	1.19E+08	33.2	7.17E+06	100	1
Superdex 200 10/300 GL	2	6.05E+07	1.21E+08	0.21	2.88E+08	102	40
Concentrated virus	0.25	1.44E+08	3.60E+07	0.42	3.43E+08	30	48
							
Hemolymph	7	2.91E+08	2.04E+09	24.2	1.20E+07	100	1
Sephacryl S-1000	30	2.40E+07	7.21E+08	0.024	1.00E+09	35	83
Concentrated virus	0.5	1.60E+08	8.00E+07	0.073	2.19E+09	4	183

As an alternative column, Sephacryl S-1000 SF column chromatography was used for rBmNPV-hPRR purification. Sephacryl S-1000 SF is optimized for the purification of DNA, viruses and spherical particles up to 400 nm and its fraction range is from 5 × 10^5 ^to 1 × 10^8 ^Da. Larval hemolymph was pretreated as mentioned above. Seven ml of larval hemolymph (2.91 × 10^8 ^pfu/ml) was applied to Sephacryl S-1000 SF column equilibrated with PBS. Elution was performed by PBS and every 5 ml fraction was collected as shown in Figure 3C. The first peak was observed between 100 and 130 ml, followed by the increasing second peak from elution volume of 200 ml. GP64 was detected in the first peak (Fraction 21–23 in Figure 3C). The maximum baculovirus number was 3.21 × 10^8 ^pfu in the fraction 22 (Figure 3D), and total number of rBmNPV-hPRR recovered was 7.21 × 10^8 ^pfu. Recovery yield of rBmNPV-hPRR was 35%, and decreased to 4% by concentration via ultracentrifugation (Table 2). rBmNPV-hPRR was finally purified by 183-fold using Sephacryl S-1000 SF column chromatography. Transfiguracion et al. [13] reported that low recovery using Sephacryl S-1000 SF column chromatography and concentration by ultracentrifugation would be improved by the treatment of hemolymph with Benzonase and buffer exchange during the purification steps involved.

Proteins of rBmNPV-hPRR purified by Superdex 200 was analyzed by SDS-PAGE which showed two bands between 60 and 80 kDa similar to hemolymph (lane 2 in Figure 3E). However, when Sephacryl S-1000 was used, these two bands disappeared (lane 3 in Figure 3E), resembling the pattern of protein bands found for rBmNPV-hPRR purified from Bm5 cell culture supernatant. Major proteins between 60 and 80 kDa, present in larval hemolymph, was removed completely. This shows that Sephacryl S-1000 holds a good performance in purification of the rBmNPV-hPRR from silkworm larval hemolymph directly, without any contaminants.

### Purification efficiency of baculovirus from larval hemolymph harvested at different postinjection time

The baculovirus-infected silkworm larvae became black in color at 4 days p.i. and displayed wandering characteristics from 4 – 5 days p.i.. After this stage, they began to die. The larval hemolymph was harvested from rBmNPV-hPRR-injected silkworm larvae (around 25 in number) at 4, 4.5 and 5 days p.i. It was then centrifuged, and filtered by 0.2 μm membrane filter to remove insoluble materials. The final harvested volume at 4, 4.5 and 5 days p.i. was 16, 16, and 12 ml, respectively. rBmNPV-hPRR was purified from hemolymph corresponding to each day by Sephacryl S-1000 SF column chromatography (Table 3). At 5 days p.i., the baculovirus concentration was 8.29 × 10^8 ^pfu/ml and protein concentration was 43.2 mg/ml of hemolymph, which were the highest among the three hemolymph samples. At 5 days p.i., purified baculovirus number (2.94 × 10^8 ^pfu) was also the highest, but purification efficiency (39 fold) was lower than that (264 fold) of hemolymph at 4 days p.i.. The number of rBmNPV-hPRR/mg protein was 1.55 × 10^9 ^pfu/mg at 4 days p.i., which was the purest among the three samples.

**Table 3:** Summary of baculovirus purification from hemolymph for 25 number of silkworm larvae harvested at different post-injection

Harvesting time (p.i.)	Volume (ml)	Titer (pfu/ml)	Virus (pfu)	Protein (mg/ml)	Virus/protein (pfu/mg)	Recovery (%)	Purification (fold)
4d							
Hemolymph	16	2.03E+08	3.25E+09	34.5	5.88E+06	100	1
Concentrated virus after Sephacryl	0.9	1.35E+08	1.22E+08	0.087	1.55E+09	4	264
4.5d							
Hemolymph	16	4.22E+08	6.75E+09	34.9	1.21E+07	100	1
Concentrated virus after Sephacryl	0.9	9.96E+07	8.96E+07	0.237	4.20E+08	1	35
5d							
Hemolymph	12	8.29E+08	9.95E+09	43.2	1.91E+07	100	1
Concentrated virus after Sephacryl	0.6	4.90E+08	2.94E+08	0.659	7.43E+08	3	39

### Analysis of rBmNPV-hPRR purified from hemolymph at 4 and 5 days post-injection

**Figure 4:**
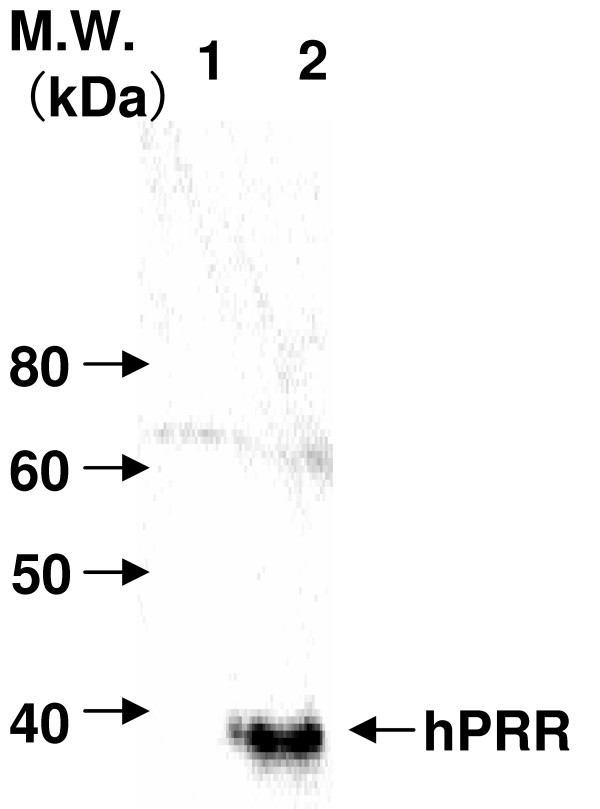
**Western blot analysis of hPRR in each purified rBmNPV-hPRR from hemolymph by Sephacryl S-1000 column chromatography**. hPRR of purified rBmNPV-hPRR was detected on reduced condition in Western blotting with mouse anti-FLAG M2 antibody, because the hPRR is a native form with FLAG peptide sequence. Lane 1, purified rBmNPV-hPRR at 4 days p.i.; lane 2, purified rBmNPV-hPRR at 5 days p.i..

To confirm the expression of hPRR on the surface of rBmNPV-hPRR, Western blot was carried out. The hPRR was observed in purified rBmNPV-hPRR at 5 days, but not at 4 days (Figure 4). Forty two nanograms of hPRR, by densitometry analysis, were obtained from 1 μg of purified rBmNPV-hPRR at 5 days p.i.. Quantity of rBmNPV-hPRR per mg of protein decreased at 5 days compared to 4 days because of an increase of protein concentration in purified rBmNPV-hPRR at 5 days.

**Figure 5:**
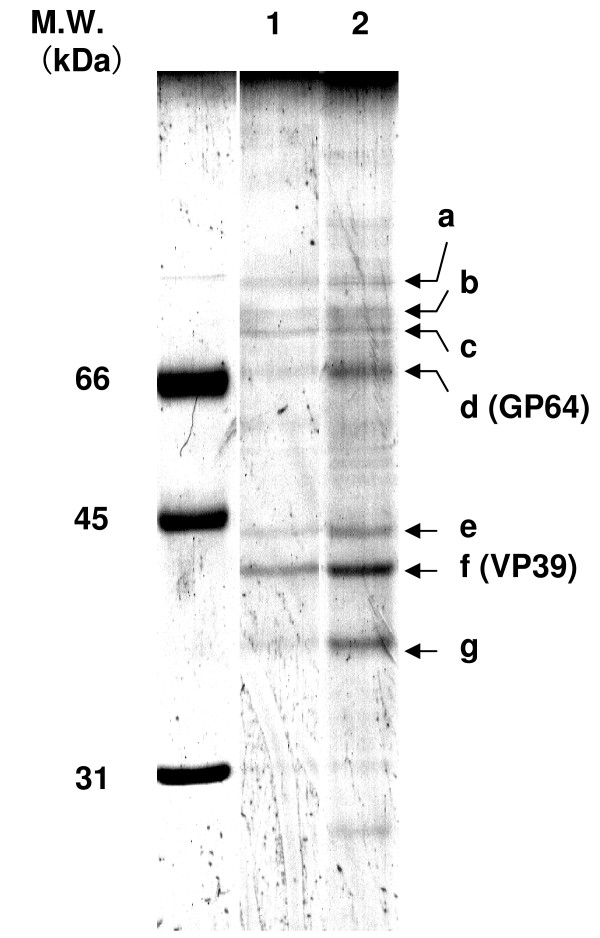
**SDS-PAGE analysis of proteins in purified rBmNPV-hPRR at 4 and 5 days by CBB stain**. Envelope and nucleocapsid proteins of purified rBmNPV-hPRR were separated by SDS-PAGE using 12% polyacrylamide. Lane 1, purified rBmNPV-hPRR at 4 days (1.1 μg of protein); lane 2, purified rBmNPV-hPRR at 5 days (1.6 μg of protein). a-g denotes main protein bands of rBmNPV-hPRR.

To analyze proteins in each purified rBmNPV-hPRR, SDS-PAGE was performed and the gel was stained with CBB (Figure 5). Pattern of protein bands was not different between purified rBmNPV-hPRR at 4 days and 5 days p.i.. This suggests that an increase of protein concentration in purified rBmNPV-hPRR at 5 days p.i. was not due to the contamination of intracellular proteins in purified rBmNPV-hPRR. Ratio of GP64 (d), a major envelope protein, to VP39 (f), a major nucleocapsid protein increased at 5 days p.i. (Table 4). Two protein bands (e and g) also increased likely with an increase in GP64. It was reported that these two bands, e and g, is the budded baculovirus-specific proteins [21]. This indicates that envelope proteins increase in rBmNPV-hPRR as infection time passes.

**Table 4:** Densitometry analysis of each protein band in purified rBmNPV-hPRR at 4 and 5 days p.i. as shown in Figure 4

Harvesting time (p.i.)	a/f	b/f	c/f	d/f	e/f	f/f	g/f
4 d	0.28	0.34	0.55	0.46	0.32	1	0.4
5 d	0.36	0.41	0.54	0.88	0.55	1	0.7
							
5 d/4 d	1.29	1.2	0.98	1.91	1.66	100	1.75

**Figure 6:**
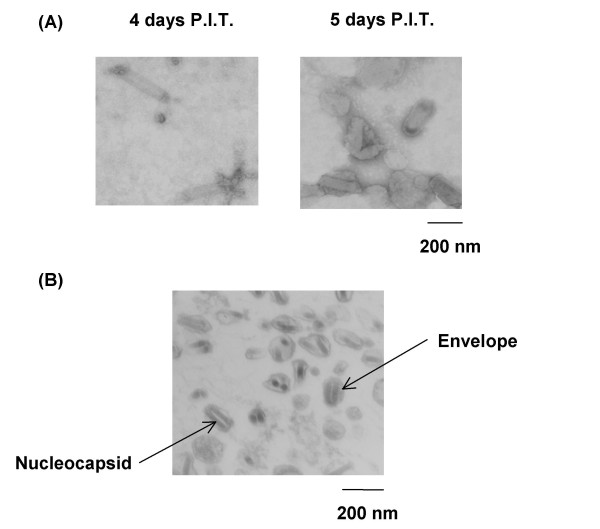
**Shapes of purified rBmNPV-hPRR from larval hemolymph at 4 and 5 days p.i**. (A) TEMs of purified rBmNPV-hPRR from hemolymph at 4 and 5 days p.i.. (B) Thin-section TEM of purified rBmNPV-hPRR from hemolymph at 5 days p.i..

When the purified rBmNPV-hPRR particles from hemolymph were observed using transmission electron microscopy (TEM), it contained aberrant sized particles (small and thick particles) at 5 days p.i. compared to that of 4 days p.i. (Figure 6A). rBmNPV-hPRR particles enclosed with thick envelopes also appeared. When thin-section TEM was performed, thick envelopes around rBmNPV-hPRR particles were observed too (Figure 6B). Moreover, envelope proteins in rBmNPV-hPRR from hemolymph were increased at 5 days p.i.. This reveals that low purification efficiency of rBmNPV-hPRR from hemolymph at 5 days p.i. might be due to thick envelope proteins formed around the rBmNPV-hPRR particles.

## Discussion

Q-PCR is applicable for the rapid titer determination of viruses like adenoviruses, retroviruses and AcMNPV [19,22-24]. Especially in the case of AcMNPV, SYBR Green I was adopted for Q-PCR and a correlation between titers determined by end-point dilution and Q-PCR has been observed. However, in the case of BmNPV titer determination, the method was dependent only on conventional plaque assay and end-point dilution method only, until now. In this study, we established the Q-PCR titer determination method for BmNPV. The melting point of amplified fragment from BmNPV DNA was 82°C, but the melting point of amplified fragment from AcMNPV DNA was approximately 86°C. Identity in DNA sequence between the two fragments is 95.8%. The difference between two melting points is supposed to result from the difference between the nucleotide sequences of the respective two fragments.

The baculovirus number from the hemolymph of a BmNPV bacmid-injected silkworm larva was 2.8 × 10^4 ^times higher than that of transfection solution in spite of usage of the same amount of BmNPV bacmid. Baculovirus infection was not observed using transfection solution as baculovirus solution, indicating that it is difficult for the amount of rBmNPV-hPRR in transfection solution to infect Bm5 cells in normal condition due to a very small amount of baculovirus titer. This implies that many amplification steps are a prerequisite towards a successful infection of Bm5 cells. It takes at least a month to amplify baculoviruses up to a sufficient titer. Using hemolymph as a baculovirus stock to increase baculovirus titer can thus save time, cost and labor. In this report, the maximum baculovirus titer of hemolymph from BmNPV-infected silkworm larvae was found to be 4.50 × 10^9 ^pfu/ml.

Sephacryl S-1000 SF column is more appropriate for the purification of BmNPV from larval hemolymph than Superdex 200 10/300 GL column, because of its efficiency in removal of contaminant proteins. In the case of purification of Lily symptomless virus from fresh infected-tissues of Lanzhou lily, Sephadex-200 HR was superior to Sephacryl S-1000 method [25]. In this study, Sephadex-200 HR also was found to be superior to Sephacryl S-1000 SF column chromatography in the baculovirus recovery, but purification yield was one-third to that with Sephacryl S-1000 SF column chromatography. Turkey coronavirus was also purified by Sephacryl S-1000 SF column chromatography from intestines and intestinal contents of infected turkey embryos [26].

Several researchers have reported that BmNPV was purified from silkworm pupae by sucrose gradient centrifugation [27,28]. In the case of purification of BmNPV from silkworm larval hemolymph, this paper gives the first report using SEC. Quantity of rBmNPV-hPRR per mg of protein decreased at 5 days p.i. compared to 4 days p.i.. However, protein-band pattern in purified rBmNPV-hPRR at 4 and 5 days p.i. was similar; this denotes that there was no contamination in purified rBmNPV-hPRR at 5 days p.i.. But proteins of which rBmNPV-hPRR is composed of may increase at 5 days p.i.. In fact, purified rBmNPV-hPRR from hemolymph at 5 days p.i. contained more envelope proteins than that at 4 days p.i.. Moreover, hPRR which is expressed in the envelope was observed at 5 days p.i., but not 4 days p.i. Electron micrographs revealed that purified rBmNPV-hPRR from hemolymph at 5 days p.i. possessed thick envelopes, resulting in low purification efficiency of rBmNPV-hPRR from hemolymph at 5 days p.i.. The increase of envelope proteins in rBmNPV-hPRR of larval hemolymph at 5 days p.i. was caused by thick envelopes around rBmNPV-hPRR particles. Besides, it was previously reported that increase of mutant virus, defective interfering baculoviruses (DIs), which lacks considerable parts of its genome, was observed during serial passage of AcMNPV in insect cells [29,30] and the large deletion of its genome may be caused by several hetero- and homologous recombination mechanism [31]. DIs of BmNPV was also found in cultured cells [32]. DIs might not be detected by Q-PCR titer measurement method because of the large deletion of its genome, probably the loss of *ie-1 *gene. It is suggested that DIs, which missed *ie-1 *gene, increased in hemolymph during BmNPV infection and therefore quantity of rBmNPV-hPRR per mg of protein decreased at 5 day p.i.

## Conclusion

The applicability of Q-PCR for BmNPV titer determination was found successful in the current study. Hemolymph from silkworm larva infected by rBmNPV (rBmNPV-hPRR) contains its titer in higher amount of BmNPV, even though 1:10^5 ^diluted hemolymph was injected to silkworm larva. Moreover, a higher titer of rBmNPV-hPRR solution (4.50 × 10^9 ^pfu/ml) was obtained from silkworm larval hemolymph. Purification of rBmNPV-hPRR from silkworm larval hemolymph was successfully implemented by Sephacryl S-1000 SF column chromatography. The purified rBmNPV-hPRR showed that the hPRR was displayed on the surface of rBmNPV. This system allows a large-scale production and purification of rBmNPV displaying recombinant protein, which can be applicable for the functional analysis of receptors, drug delivery system and vaccines against infectious viruses and protozoa.

## Methods

### Insects

For silkworm infection, hemolymph or transfection solution of recombinant baculovirus-infected larvae was injected into the first day of fifth instar larvae (Ehime Sansyu Co. Ltd., Ehime, Japan). After 3–5 days, silkworm larval hemolymph was collected.

### Construction of rBmNPV-hPRR bacmid

hPRR is a native form with FLAG peptide sequence behind its signal peptide sequence, as reported previously [33]. *hPRR *was amplified by PCR using prorenin-F (CACCATGGCTGTGTTTGTCGTGCTCCTGGCGTTGGTGGCGGGTGTTTTGGGGGACTACAAGGACGACGACGACAAG) and prorenin-R (ACGGAATTCTAATCCATTCGAATCTTCTGG) primers and inserted into pENTR/D-TOPO by TOPO cloning method (Invitrogen, Carlsbad, CA, USA). The details of DNA amplification cycle are: 95°C for 3 min for one cycle, followed by 30 cycles of amplification with denaturation at 95°C for 30 s, annealing at 50°C for 30 s and the final extension at 72°C for 2 min. rBmNPV-hPRR bacmid was constructed using *Escherichia coli *BmDH10Bac [34], which was transfected in Bm5 cells. After 6 days cultivation, rBmNPV-hPRR bacmid was harvested.

### Titer determination of BmNPV particles by quantitative real-time PCR

Baculoviral DNA was extracted by High Pure Viral Nucleic Acid Kit (Roche Diagnostics K. K., Tokyo, Japan) according to the manufacturer's protocol. The titration assay using Q-PCR was performed by Mx3000P system (Stratagene, La Jolla, CA, USA). The ie-1-specific primers, Bmie-1-F (CCCGTAACGGACCTTGTGCTT) and Bmie-1-R (TTATCGAGATTTATTTACATACAACAAG) were used. For the Q-PCR assays, FullVelocity SYBR Green QPCR Master Mix (Stratagene) was used. A 6 μl of extracted baculoviral DNA was used for Q-PCR in a 25 μl of final reaction mixture containing 12.5 μl of 2 × FullVelocity SYBR Green QPCR master mix, 1:500-diluted reference dye, and 0.1 μM Bmie-1-F and Bmie-1-R primers. Program for DNA amplification cycle was: 95°C for 30 s for one cycle, followed by 80 cycles of amplification protocol: denaturation at 95°C for 10 s, annealing and extension at 60°C for 30 s. PCR amplification and melting curves were analyzed by MxPro software (Stratagene).

### Production and purification of BmNPV from larval hemolymph by Superdex 200 10/300 GL or Sephacryl S-1000 SF column chromatography

Production of rBmNPV-hPRR in silkworm larvae was performed by injecting 30 μl of 100-fold phosphate buffered saline (PBS)-diluted hemolymph into silkworm larvae at second day of fifth instar followed by breeding for 4–5 days. Hemolymph from BmNPV-infected silkworm larvae was centrifuged at 20000 × g for 10 min. The collected supernatant filtered with 0.2 μm filter is then applied to Superdex 200 10/300 GL column (1.0 × 24 cm, GE Healthcare UK Ltd., Buckinghamshire HP7 9NA, England) or Sephacryl S-1000 SF column (2.6 × 52 cm, GE Healthcare UK Ltd.) equilibrated with PBS (pH 6.2). Elution was performed at 4°C chromate-chamber and monitored by absorbance at 280 and 254 nm. Every 0.5 or 5 ml of fraction was collected in the case of Superdex 200 10/300 GL column or Sephacryl S-1000 SF column. Fractions containing BmNPV particles were pooled and then were concentrated by ultracentrifugation at 114000 × g with 25% sucrose cushion (25% sucrose in 5 mM NaCl and 10 mM EDTA). Particles were suspended with a small volume of PBS (pH 6.2).

### SDS-PAGE and Western blot

Proteins were separated by sodium dodecyl sulfate-polyacrylamide gel electrophoresis (SDS-PAGE) using 12% polyacrylamide and subjected to Western blot. Under the non-reducing condition, samples were mixed with sample buffer lacking β-mercaptoethanol and boiled. Following it, the proteins were blotted onto a polyvinylidene fluoride (PVDF) membrane using Mini Trans-Blot Electrophoretic Transfer Cell (Bio-Rad, Hercules, CA, USA). After being blocked in 5% skim milk in Tris-buffered saline containing 0.1% Tween 20 (TBST), the membrane was incubated in either 1:10000 diluted mouse anti-FLAG M2 antibody (Sigma-Aldrich, St. Louis, MO, USA) or 1:4000 diluted rabbit anti-Bmgp64 polyclonal antibody for 1 hour. The membrane was washed with TBST, and then incubated in 1:20,000 diluted anti-mouse or anti-rabbit IgG antibody labeled with horseradish peroxidase for 1 hour. Detection was performed using ECL Plus Western blotting reagent (GE Healthcare UK Ltd.). Specific bands were detected using a Fluor-S/MAX multi-imager (Bio-Rad). Bands on SDS-PAGE gels were analyzed by Quantity One 1-D analysis software (Bio-Rad).

### Transmission electron microscopy (TEM)

BmNPV particles were fixed with a mixture of 2% paraformaldehyde and 2% glutaraledehyde in 0.1 M cacodylate buffer, pH 7.4 and postfixed in 1% osmium tetroxide in the same buffer. After further washes with the above buffer, the specimens were collected and embedded in agarose. The agarose blocks were dehydrated in ethanol and embedded in an Epon/Araldite mixture. The ultrathin sections were stained with uranyl acetate and lead citrate, and then examined with a Hitachi H7500 electron microscope at 80 kV.

## Authors' contributions

TK carried out the experimental design, the molecular genetic and biochemical experiments. SLM participated in rearing silkworm and sample collection from silkworm. ST participated in TEM experiments. EYP directly supervised the project, participated in its experimental design, data interpretation, and was responsible for writing the manuscript. All authors have read and approved the manuscript.
